# FARE-CAFE: a database of functional and regulatory elements of cancer-associated fusion events

**DOI:** 10.1093/database/bav086

**Published:** 2015-09-16

**Authors:** Praveen Kumar Korla, Jack Cheng, Chien-Hung Huang, Jeffrey J. P. Tsai, Yu-Hsuan Liu, Nilubon Kurubanjerdjit, Wen-Tsong Hsieh, Huey-Yi Chen, Ka-Lok Ng

**Affiliations:** ^1^Department of Bioinformatics and Medical Engineering, Asia University, Taichung 41354, Taiwan,; ^2^Graduate Institute of Integrated Medicine, College of Chinese Medicine, China Medical University, Taichung 40402, Taiwan,; ^3^Department of Computer Science and Information Engineering, National Formosa University, Yunlin 632, Taiwan,; ^4^School of Information Technology, Mae Fah Luang University, Chiang Rai 57100, Thailand,; ^5^Department of Pharmacology, China Medical University, Taichung 40402, Taiwan,; ^6^Department of Obstetrics and Gynecology, China Medical University Hospital, China Medical University, Taichung 40402, Taiwan, and; ^7^Department of Medical Research, China Medical University Hospital, China Medical University, Taichung 40402, Taiwan

## Abstract

Chromosomal translocation (CT) is of enormous clinical interest because this disorder is associated with various major solid tumors and leukemia. A tumor-specific fusion gene event may occur when a translocation joins two separate genes. Currently, various CT databases provide information about fusion genes and their genomic elements. However, no database of the roles of fusion genes, in terms of essential functional and regulatory elements in oncogenesis, is available. FARE-CAFE is a unique combination of CTs, fusion proteins, protein domains, domain–domain interactions, protein–protein interactions, transcription factors and microRNAs, with subsequent experimental information, which cannot be found in any other CT database. Genomic DNA information including, for example, manually collected exact locations of the first and second break points, sequences and karyotypes of fusion genes are included. FARE-CAFE will substantially facilitate the cancer biologist’s mission of elucidating the pathogenesis of various types of cancer. This database will ultimately help to develop ‘novel’ therapeutic approaches.

**Database URL:** http://ppi.bioinfo.asia.edu.tw/FARE-CAFE

## Introduction

Chromosomal rearrangements play a crucial role in the progression of cancer, and in particular, in chromosomal translocation (CT) events; their corresponding fusion genes (FGs) are essential in the initiation and/or development of cancer (1). FGs are frequently examined in clinical diagnosis, treatment and cancer prognosis (2). FG events account for at least 20% of all cancer cases (3).

In the past two decades, commonly used cytogenetic techniques (FISH, SKY, CGH and PCR) and recent advances in sequencing technologies have revealed a high number of CT events in human tumors (4). These experimental discoveries have improved our understanding of the pathogenetic importance of CT events in carcinogenesis (5, 6).

Mitelman’s database (7), dbCRID (4), TICdb (8), HYBRIDdb (9) and ChimerDB (10) are databases of cancer-associated CTs and FGs that have been identified experimentally and in the literature. The four databases other than Mitelman’s database provide fusion sequences with the positions of break points at nucleotide level. Only dbCRID provides the ‘exact’ positions of break points, and it does so for only a ‘few’ FGs; in contrast, this work provides ‘exact’ break point information for ‘all’ FGs including their ‘isoforms’. Break point positions are mapped using the Human genome assembly hg19 (NCBI Build 37.1 Feb 2009).

During transcription, transcription factors (TFs) regulate the promoter region of the FG. These TFs enhance or inhibit the transcription of the tumorigenic FGs. At the post-transcriptional modification level, the expression of the fusion transcript that was regulated by microRNAs (miRNAs) was studied. These miRNAs may target the 3′-end of the FG, suppressing the expression of the FG, and thereby promote apoptosis and reduce cell proliferation (11, 12). In summary, these two regulatory elements, TFs and miRNAs, crucially affect cancer progression.

**Figure 1.**
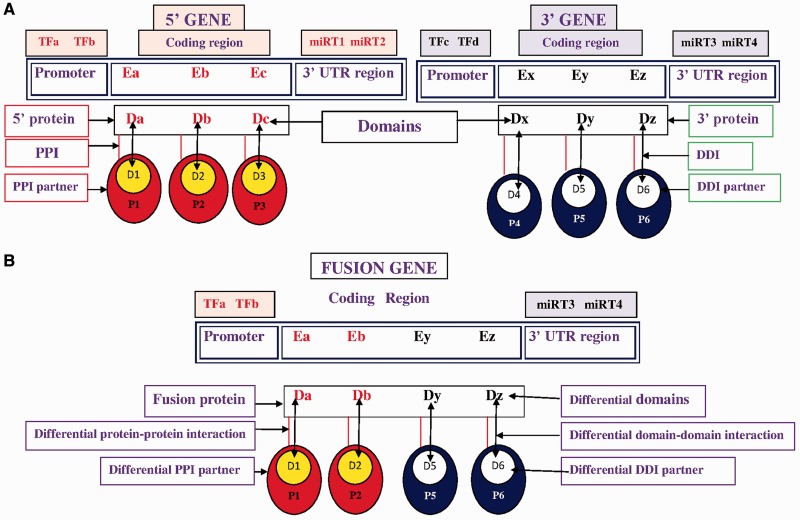
Pictorial representation of TFs, miRNA target, differential domains, DDIs and PPIs in FG. (**A**) Wild-type 5′ gene and 3′ gene with their TFs, miRNAs, domains, DDIs and PPIs. (**B**) FG associates with a different set of (i) TFs, i.e. TFa and TFb; (ii) miRNA targets (miRT) at 3′-UTR region, i.e. miRT3 and miRT4; (iii) domains denoted by Da, Db, Dy and Dz; (iv) DDIs denoted as Da-D1, Db-D2, Dy-D5 and Dz-D6; and (v) DDI-mediated PPI partners, i.e. P1, P2, P5 and P6.

Based on the first and second break points of FG, the coding regions were extracted and the domain composition of the fusion proteins is annotated. 5′ and 3′ partner genes of FG have fewer domains than wild-type 5′ and 3′ genes due to translocation, so the FGs failed to exhibit certain domain–domain interactions (DDIs) after translation. The absence of these domains disrupted certain DDIs and DDI-mediated protein–protein interactions (PPIs) (Figure 1), further disrupting the regular biological processes that are associated with cancer-associated pathways.

In this work, a number of resources, including FGs, TFs, miRNAs, miRNA targets, domains, information about DDIs and PPIs are integrated to construct a database of functional and regulatory elements of cancer-associated fusion events (FARE-CAFE). This database supports an examination of the role of cancer-associated FGs/proteins at various levels of regulation, which are transcription initiation (TF￫FG), post-transcriptional regulation (miRNA￫FG) and functional interaction corresponding to domains, DDIs and PPIs. To the best of our knowledge, this database is the first to address the relationships among FGs, TFs, miRNAs, DDIs and PPIs in a study of cancer (Figure 1).

## FARE-CAFE database

### Input data

**Figure 2.**
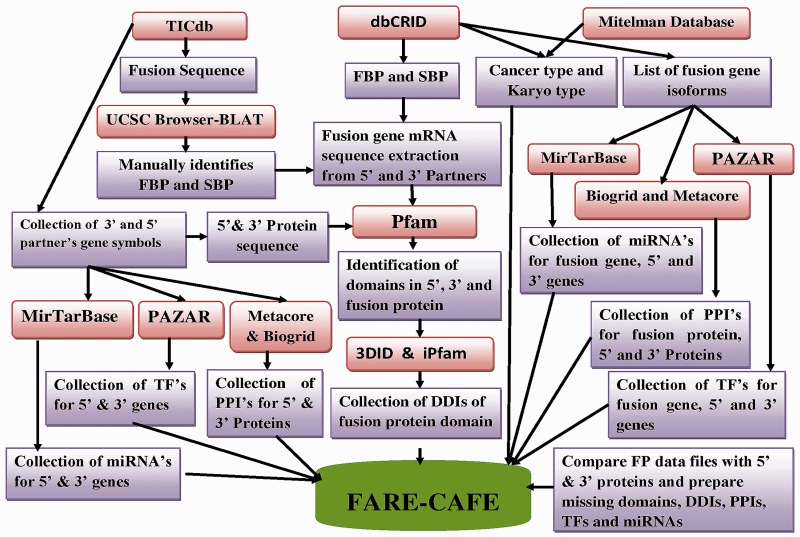
FARE-CAFE methodology workflow.

To construct FARE-CAFE, functional, regulatory and genomic information about FGs were collected from various data sources (Supplementary Table S1). Figure 2 presents the workflow of FARE-CAFE. Genomic information about the FGs was obtained from Mitelman’s database, dbCRID and TICdb. Positions of break point at nucleotide level in fusion sequences were obtained from dbCRID and TICdb. Data concerning functional elements, such as domains from Pfam (13), DDIs from 3DID (14), PPIs from both of BioGrid (15) and Metacore, were also used. With respect to regulatory elements, TFs are obtained from PAZAR (16), miRNA targets from MirTarBase (17), karyotypes and types of cancer from OMIM (18), and mRNA and protein sequences from the Refseq database. The first break point (FBP) and second break point (SBP) in the exon/intron are retrieved from the UCSC genome browser (19) using the pairwise alignment tool, BLAT (20). Information about sub-cellular localization and the tissue specificity of wild-type proteins is obtained from Swiss-Prot (21). Table 1 presents statistics concerning the input data.

**Table 1.** FARE-CAFE database statistics

FARE-CAFE Database	Number of entities
Total number of FGs	518
Total number of FG isoforms	1587
Total number of 5′ gene partners	270
Total number of 3′ gene partners	295
Major types and sub-types of cancers	122

### Implementation

FARE-CAFE provides a user-friendly interface that can be utilized by researchers to search for cancer-specific fusion events and information about their functional and regulatory elements, recorded using MySQL. The graphical web interface was built using the PHP language.

### Query interface

FARE-CAFE provides a web-based interface with which users query and access information about cancer-specific FGs. Query keywords include cancer types, FGs, names of 5′ proteins and names of 3′ proteins, all of which can be selected using a pull down menu. For instance, if the user selects an FG as a query option, then the database will return cancer types, genomic, functional, regulatory and sequence features on the output page. The user can search on keywords that specify cancer type, or 5′ and 3′ gene names to access cancer-associated data and information about wild-type proteins functional (domains, DDIs and PPIs) and regulatory elements (TFs and miRNA targets). Apart from the search page, the other web pages provide essential information, such as database statistics, pictorial representations of the workflow, TFs, miRNAs, domains, disrupted domains, DDIs, PPIs, case studies and downloadable files that are related to the FGs in the FARE-CAFE database.

### Case studies

To demonstrate the usefulness of FARE-CAFE in identifying the role of regulatory and functional elements of FGs in cancer progression, three case studies are examined; these case studies will increase the impacts of our database in cancer biology research.

## Results and discussion

The FARE-CAFE database includes a comprehensive collection of FGs, CT events and their essential genetic elements which are necessary to understand the mechanism of cancer progression, and are not provided by most existing databases. FARE-CAFE includes 518 FGs, 1587 FG isoforms, 122 major types and subtypes of cancers, 270 five-prime proteins and 295 three-prime proteins annotated with key genetic elements that are involved in cancer. All existing CT-related databases provide only gene level or partial genomic level information. Each of these databases has distinct strengths and uses. A comparison of FARE-CAFE to other database resources is given in Table 2.

**Table 2.** A comparison of FARE-CAFE and other related databases

Features	TICdb	ChimerDB	dbCRID	FARE-CAFE
FGs with break points information	**√**	**√**	**√**	**√**
Fusion sequence/Junction sequence	**√**	**√**	**√**	**√**
FGs identified methods documented	**—**	**—**	**√**	**√**
Manually identified exact break point positions	**—**	**—**	Partially	**√**
Cancer type information	**—**	**√**	**√**	**√**
List of domains in fusion protein	**—**	**—**	**—**	**√**
DDIs of fusion protein domains	**—**	**—**	**—**	**√**
PPIs of fusion protein	**—**	**—**	**—**	**√**
5′ and 3′ partner proteins domains, DDIs and PPIs information	**—**	**—**	**—**	**√**
Differential miRNAs at FG—3′-UTR	**—**	**—**	**—**	**√**
Differential TF at FG—5′-end	**—**	**—**	**—**	**√**
FG break points located in exon or in intron region documented	**—**	**—**	**—**	**√**
5′ and 3′ proteins sub-cellular localization and tissue specificity	**—**	**—**	**—**	**√**

**Figure 3.**
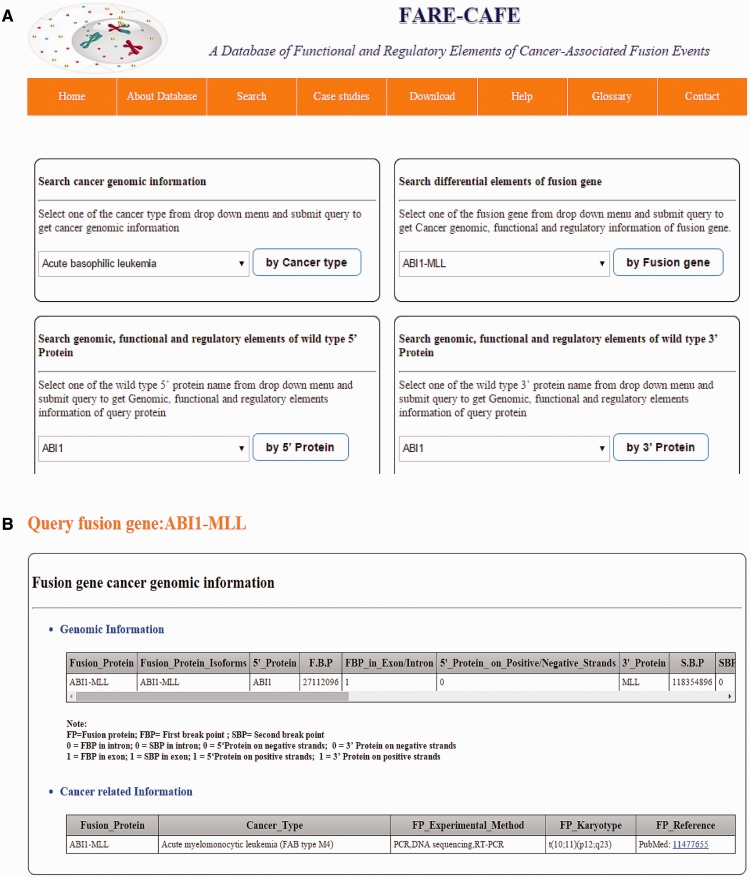

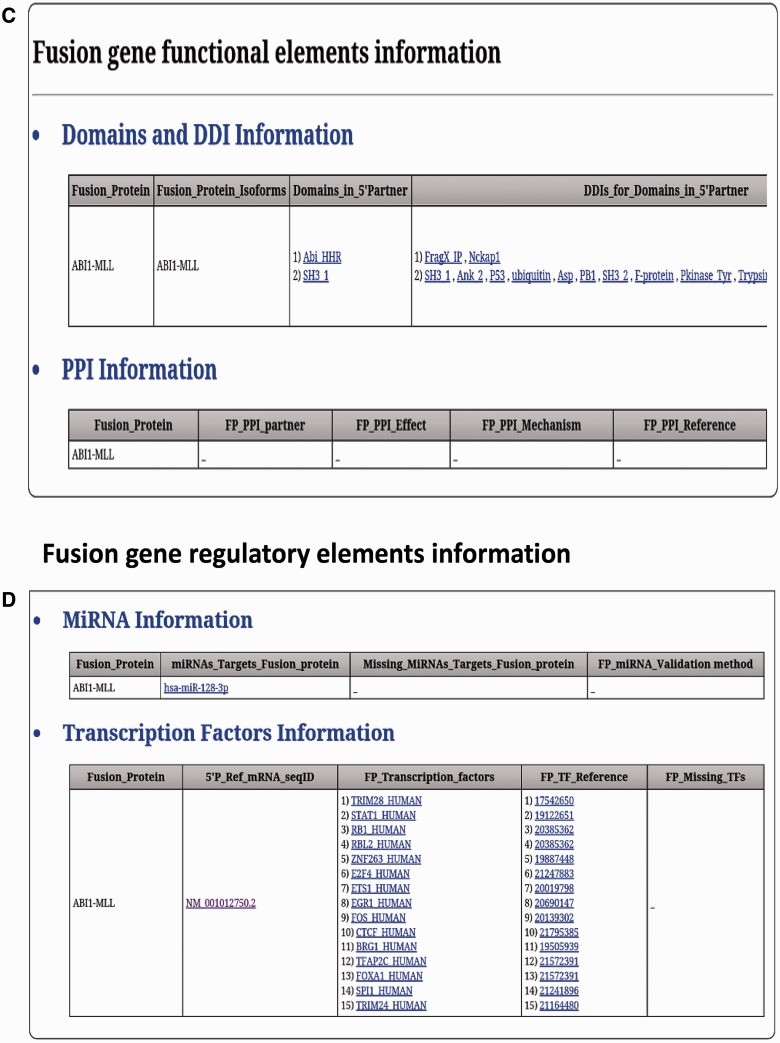
FARE-CAFE search and result page. (**a**) Search for ABI1-MLL fusion gene. (**b**) Result page with cancer genomic elements information of ABI1-MLL fusion gene. (**c**) Result page with functional elements information of ABI1-MLL fusion gene. (**d**) Result page with regulatory elements information of ABI1-MLL fusion gene.

FARE-CAFE is the only database that provides information about TFs, miRNAs, domains, DDIs and PPIs for FGs. A snapshot of the search and result pages of FGs is depicted in Figure 3. The missing TF, miRNA, domain and DDI components of FGs are determined by comparison with wild-type 5′ and 3′ gene partners. The significance of the presented database is demonstrated using three case studies, which illustrate the role of the regulatory elements and functional elements in cancer formation by CTs.

### Case study 1: Impact of TFs on FG-induced cancer progression

**Figure 4.**
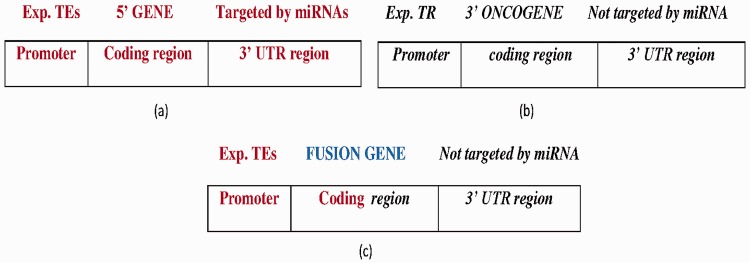
(**a**) The wild-type 5′ gene is activated by experimentally reported transcription enhancers (Exp.TEs) at the promoter region and with experimentally reported targeted miRNAs at the 3′ UTR region. (**b**) The wild-type 3′ oncogene inhibited by experimentally reported transcription repressor (Exp. TR) at the promoter region and not targeted by miRNA at the 3′-UTR region. (**c**) The FG composed of 5′ gene transcribed by TEs at the promoter region, and coding region forms from the fusion of the 5′ gene and 3′ oncogene which is not targeted by miRNA at the 3′-UTR region. Bold-faced and italic fonts denote the wild-type 5′ gene and 3′ gene, respectively.

FGs with the following features were collected: (i) the 5′ gene was transcribed by TFs (using TF information from the PAZAR database and activity information from Metacore database) (Figure 4a), (ii) the 3′ gene is oncogenic (from NCG 4.0) (22), (iii) the 3′ gene expression is controlled by a transcription repressor (from the Metacore database) and (iv) the 3′ gene is not targeted by miRNAs (Figure 4b). Figure 4c displays the FG structure. A total of 14 FGs satisfy the specified constraints (Supplementary Table S2).

### Observations and analysis

- The FG is transcribed by various TFs, which bind the 5′ gene promoter region.
- After translocation, the 3′ oncogene lost its TF binding sites for the transcription repressor and fused with the 5′ gene.
- miRNA does not target the 3′ partner gene of the FG (e.g. *BCL2-IGH*), so the 3′ gene-specific transcription repressor or miRNA does not suppress this kind of FG (e.g. all FGs reported in this case study). The *ETS1* (from Metacore) acts as a transcription repressor for the wild-type 3′ gene but a transcription enhancer for two FGs in which *ETS1* activates the 5′ gene—*PAX5-JAK2* (23, 24) and *RUNX1-CBFA2T3* (25, 26). Another TF, EBF, is found to act on the FG, *PAX5-ZNF521* (23). Here, the EBF activates the wild-type 5′ gene *PAX5*, but represses the wild-type 3′ gene *ZNF521* (from Metacore).

In summary, owing to CT, the wild-type 3′ oncogene lost its transcription inhibition site and fused with the 5′ gene. This FG (all FGs reported in this case study) may exhibit oncogenic characteristics after translocation. The 3′ gene fragment does not lose its oncogenic nature because it utilizes the 5′ gene promoter for transcription.

### Case study 2: Impact of miRNAs on FG-induced cancer progression

**Figure 5.**
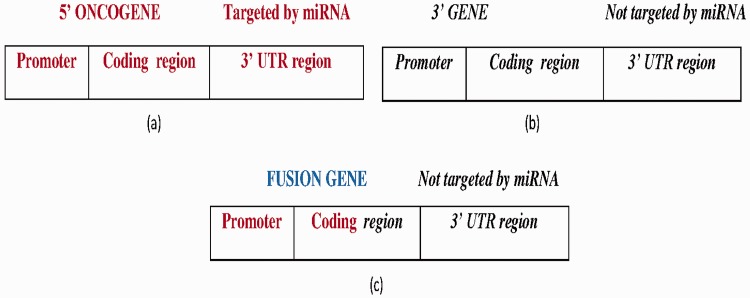
(**a**) The wild-type 5′ oncogene targeted by experimentally reported miRNAs at the 3′-UTR region. (**b**) The wild-type 3′ gene not targeted by any miRNA. (**c**) The FG with a 5′ oncogene promoter, coding region fragment forms from the fusion of the 5′ oncogene and 3′ gene, where the 3′ gene is not targeted by any miRNA. Bold-faced and italic fonts denote the wild-type 5′ gene and 3′ gene, respectively.

FGs with the following two features were obtained from miRTarbase and NCG 4.0: (i) the 5′ gene is oncogenic with experimentally verified miRNAs that are targeted at its 3′ UTR (Figure 5a) and (ii) the 3′ gene is not targeted by any miRNA (Figure 5b). To address feature (ii), three miRNA target prediction algorithms—miRDB (27), MiRanda (28) and TargetScan (29)—were utilized to determine whether the 3′ UTR of the 3′ gene was targeted by any miRNA or not. Then, the predicted miRNAs were compared with the miRNAs of the 5′ gene. If any one of the predictions made using the algorithm matched the 5′ gene miRNA, then a score (*S*) of 1 was assigned to the prediction, so the maximum score was 3. To improve the reliability of the prediction, the predicted miRNAs were predicated against the miRCancer database (30) to determine whether those miRNAs had been recorded. MiRCancer is a database of cancer-related miRNAs. Table 3 summarizes the four possible outcomes of this process.

**Table 3.** The four possible outcomes based on the results of score *S* and miRCancer

Score, *S*	miRNA found in miRCancer	Hypothesis
*S* ≥ 1	No	miRNA has a low possibility of regulating the FG
*S* ≥ 1	Yes	miRNA has high possibility of regulating the FG
*S* = 0	No	miRNA has a very low possibility of regulating the FG
*S* = 0	Yes	miRNA has a low possibility of regulating the FG

A total of 33 FGs that satisfied the above constraints (Figure 5c) were found, about which detailed information can be found in Supplementary Table S3. Notably, if a 3′ gene is not targeted by any miRNA and its host FG is activated, then that FG is more likely to have an oncogenic role than wild-type 3′ gene.

### Observations and analysis

After CT, all of the 33 collected FGs lost the 3′-UTR regions from their 5′ oncogenes, and so were not regulated by any miRNA.

- The 5′ oncogenes fused with 3′ genes, which were not targeted by any miRNA.
- A majority (more than 95%) of the 3′ genes predicted miRNAs were not shared by the 5′genes. The remaining 5% were overlapping miRNAs.
- The predictions are supported by the findings that over 90% of the 3′ genes’ predicted miRNAs were not associated with any record in miRCancer. The other 10% miRNAs were identified in mirCancer.

Owing to CT, the 5′ oncogenes of the 33 FGs lost their miRNA sites and fused with 3′ partner genes that were not targeted by any miRNA. As the FGs did not have any accessible site that was targeted by miRNAs, those FGs may have had a key role in enhancing cell proliferation and inhibited apoptosis, which leads to cancer progression.

Our case study is strengthened by the work of Shugay *et al*.** (31). The previous work reported that 3′ translocation partner genes (TPGs) contain significantly shorter 3′-UTRs than 5′ TPGs. Hence, there were fewer regulatory elements and miRNA target sites in the 3′-UTRs of 3′ TPGs, thus disrupting FGs from miRNA-mediated gene repression (32). Gomez-Benito *et al.* (33), shown that substituting the 3′-UTR of the *MLL* gene with the 3′-UTR of its fusion partner, resulted in significantly post-transcriptional inhibition of the expression of *MLL*, which leads to *MLL*-mediated CT events.

### Case study 3: Impact of functional elements on FG-induced cancer progression

The impacts of the functional elements—domains, DDIs and PPIs—of the fusion proteins in acute myeloid leukemia (AML), chronic myeloid leukemia (CML) and Ewing’s sarcoma cancer progression are presented in the following. A total of 38 fusion proteins with 203 isoforms are associated with the three selected types of cancer. Supplementary Table S4 presents the functional elements of those fusion proteins and their wild-type 5′ and 3′ partner proteins.

### Observations and analysis

- After translocation, fusion protein, which is composed of a smaller group of domains than the union of its 5′ and 3′ wild-type genes, is formed.
- As the fusion protein is composed of a smaller set of domains, certain biologically significant DDIs and DDI-mediated PPIs are disrupted.
- For instance, the *BCR-ABL* fusion protein (which causes AML and CML) interacts with the *GAB2* (which contains a PH domain) and *H-Ras* (which contains a Ras domain) proteins via DDI with the RhoGEF domain (which contains in *BCR*). These PPIs play a key role in the ‘CML pathway’ (KEGG ID: hsa05220). Also, *BCR-ABL* interacts with the *CRK* and *STAT5A* proteins (with the SH2 domain in *CRK* and *STAT5A*) via DDI with the C2 domain (which contains in *BCR*). These PPIs also play a role in the ‘CML pathway’. However, certain *BCR-ABL* fusion isoforms (34, 35) lost the RhoGEF and C2 domains, disrupting the ‘CML pathway’.
- With respect to Ewing’s sarcoma cancer, the RRM_6 domain in protein *EWSR1* interacts with the *SF3B4* and *SRSF5* proteins; this interaction is presented in the ‘Spliceosome pathway’ (hsa03040). The PPI is mediated by the DDI between domains RRM_6 and RRM_1 (which contains in *SF3B4* and *SRSF5* proteins). Also, the zf-RanBP domain in *EWSR1* interacts with the *RAD23A* and *HERPUD1* proteins. This interaction is recorded in ‘Protein processing in endoplasmic reticulum pathway’ (hsa04141).

The PPI is mediated by the DDI between domains zf-RanBP and ubiquitin (which contains in *RAD23A* and *HERPUD1* proteins). After translocation, *EWSR1* lost both RRM_6 and zf-RanBP domains and fused with *FLI1* to form the oncogenic fusion protein *EWS-FLI1*, causing Ewing’s sarcoma. [For more details, see ‘Sarcomas’ under ‘Transcriptional Mis-regulation in Cancer’ (hsa05202).]

Relative to their 5′ and 3′ gene partners, the fusion proteins lost some essential functional elements, such as domains (Supplementary Table S4), and subsequently disrupting a few DDIs or DDI-mediated PPIs, interrupting certain biological processes that may play crucial roles in cancer formation.

Case study 3 is in compatible with the work by Shugay *et al.* Both works have certain aspects in common, for example, their work made use of protein interaction interfaces information of a TPG to list its interaction partners, whereas our work employed DDI and PPI databases to achieve this goal.

It is interesting to note that Shugay *et al.* classified all protein domains into five functional classes and studied the co-occurrence of any class using Fisher exact test. It was also found that 5′ fusion partner contains domain with non-oncogenic properties could contribute a strong promoter and 3′ fusion partner associates with oncogenic domains might stabilize the fusion mRNA by contributing its 3′-UTR.

### Future works for the FARE-CARE database

In future version of FARE-CAFE, data obtained from four databases, i.e. COSMIC (36), Mitelman (7), TCGA Fusion Gene Data Portal database (37) and ChimerDB, will be incorporated.

COSMIC is the largest and the most comprehensive resource for somatic mutations in human cancers. The Mitelman database provides huge collection of FG events and their tumor characteristics information. The TCGA Fusion Gene Data Portal database provides a list of fusion events with clinical relevance that have not been previously recognized in various cancer types. This database group detects fusion transcripts based on integrated analysis of RNA sequencing. ChimerDB is one of the large scale fusion events database with huge number of fusion transcripts annotated with mRNA and EST and NGS fusion transcripts information.

We will utilize the above-mentioned resources and include additional information in FARE-CAFE. For instance, new/novel FGs (from Mitelman database and TCGA Fusion Gene Data Portal database) with exon annotations, CDS mutation sites, mutation types, mutation frequency and tissue-specific information for FGs (from COSMIC), mRNA sequence and EST sequence (from ChimerDB, but it is not update since 2010), which are crucial for cancer diagnosis and therapy. Furthermore, FARE-CAFE will provide RNA sequencing information of cancer samples by linking to the TCGA Fusion Gene Data Portal database.

We anticipate to regularly update FARE-CAFE once new versions of the utilized databases are released.

## Summary

FARE-CAFE is the first comprehensive database of extensive annotated oncogenic FG events, including, genomic, regulatory and function elements, which play crucial roles in cancer formation. Overall, this database enables the finding of relevant information (TF, miRNA, domain, DDI and PPI) about cancer-associated FGs at various levels (pre-transcription, post-transcription and post-translation) of regulations and serves as a unique resource for researchers in the field of the cancer biology.

## Supplementary Data

Supplementary data are available at *Database* Online.

Supplementary Data
