# Supplementary material for: FARE-CAFE: a database of functional and regulatory elements of cancer-associated fusion events
Source: Database (Oxford). 2015 Sep 16;2015:bav086. doi: 10.1093/database/bav086 (PMC4684693; doi:10.1093/database/bav086)
Supplement: Supplementary Data [file supp_2015_bav086_index.html]

FARE-CAFE: a database of functional and regulatory elements of cancer-associated fusion events — Supplementary Data 

# FARE-CAFE: a database of functional and regulatory elements of cancer-associated fusion events

## Supplementary Data

files

- Supplementary Data - zip file
