# Supplementary material for: FARE-CAFE: a database of functional and regulatory elements of cancer-associated fusion events
Source: Database (Oxford). 2015 Sep 16;2015:bav086. doi: 10.1093/database/bav086 (PMC4684693; doi:10.1093/database/bav086)
Supplement: Supplementary Data [file supp_bav086_suppl_data.zip › Supplementary Table S1.doc]

**Supplementary information**

**Supplementary Table S1**

Table S1: Summary of the data resources and type of data provided by the databases used in constructing the FARE-CAFE database

| **Data resource** | **Web link** | **Type of data** |
| --- | --- | --- |
| TICdb &  dbCRID | <http://www.unav.es/genetica/TICdb/>  <http://dbcrid.biolead.org/> | Fusion genes and partners |
| NCBI | <http://www.ncbi.nlm.nih.gov/> | Fusion partner genes nucleotide and protein sequences |
| dbCRID | <http://dbcrid.biolead.org/> | Cancer types and fusion breakpoint information |
| UCSC Browser | <https://genome.ucsc.edu/> | Mapping breakpoint and Fusion sequences extraction |
| Pfam | <http://pfam.sanger.ac.uk/> | Domain information |
| 3DID&  *i*Pfam | <http://3did.irbbarcelona.org/>  <http://www.ipfam.org/> | Domain-domain interaction information |
| Metacore &  BioGRID 3.2 | <http://thomsonreuters.com/metacore/>  <http://thebiogrid.org/> | Protein-protein interaction information |
| PAZAR db | <http://www.pazar.info/> | Transcription factors information |
| miRTarBase | <http://mirtarbase.mbc.nctu.edu.tw/> | miRNA target genes information |
